# Multifunctional Xanthan Gum–Hydroxyapatite Composite with Enhanced Dye Adsorption, Antibacterial Activity, and Cytocompatibility

**DOI:** 10.3390/ma19183881

**Published:** 2026-09-11

**Authors:** Yassine Benali, Rostom Lakhdar, Daniela Predoi, Simona Liliana Iconaru, Carmen Steluta Ciobanu, Krzysztof Rokosz, Andrei Craifặleanu, Khaled Boughzala

**Affiliations:** 1 Laboratory of Materials Applications in Environment, Water and Energy LAMEEE (LAM3E), Faculty of Sciences of Gafsa, University of Gafsa, Gafsa 2112, Tunisia; yassin.bn.ali27@gmail.com (Y.B.); rostomlakhdar19@gmail.com (R.L.); 2 National Institute of Materials Physics, Atomistilor Street, No. 405A, P.O. Box MG 07, 077125 Magurele, Romania; simonaiconaru@gmail.com (S.L.I.);; 3 Faculty of Electronics and Computer Science, Koszalin University of Technology, Śniadeckich 2, 75-453 Koszalin, Poland; rokosz@tu.koszalin.pl; 4 Department of Mechanics, National University of Science and Technology POLITEHNICA Bucharest, BN 002, 313 Splaiul Independentei, Sector 6, 060042 Bucharest, Romania; ycraif@yahoo.com or andrei.craifaleanu@upb.ro; 5 Laboratory of Physical-Chemistry of Materials, Faculty of Sciences of Monastir, Avenue de l’Environnement, Monastir 5019, Tunisia

**Keywords:** hydroxyapatite–xanthan composite, dye adsorption, antibacterial properties, wastewater treatment, biomedical applications

## Abstract

Hydroxyapatite (HAp) is a promising biomaterial with potential environmental and biomedical applications; however, its surface properties and functional performance can be improved through polymer modification. In this study, a novel hydroxyapatite xanthan (HAp-XAn) composite was developed and investigated for both environmental and biomedical applications. Comprehensive structural and surface analyses were carried out using X-ray diffraction (XRD), Fourier transform infrared spectroscopy (FTIR), X-ray photoelectron spectroscopy (XPS), scanning electron microscopy (SEM), and atomic force microscopy (AFM) measurements. The biocompatibility of HAp and the HAp-XAn composite was assessed with the aid of the MG63 cell line using the MTT (3-[4,5-dimethylthiazol-2-yl]-2,5 diphenyl tetrazolium bromide) assay. The findings indicate that both HAp and HAp-XAn exhibit good in vitro cytocompatibility and favorable interactions with osteoblast-like cells, supporting their potential for use in bone-related biomedical applications. The antibacterial properties of HAp and HAp-XAn composites were studied in vitro against *Staphylococcus aureus* ATCC 25923 (*S. aureus*) and *Escherichia coli* ATCC 25922 (*E. coli*) bacterial strains. The results of the antibacterial assay revealed that HAp-XAn exhibited an improved antibacterial activity compared with HAp. The antibacterial assay highlighted that the inhibitory effect of HAp-XAn increased with the increase in incubation period. The HAp-XAn composite exhibited enhanced methylene blue (MB) adsorption compared with HAp, with the adsorption performance strongly dependent on pH, contact time, and initial dye concentration. Kinetic and equilibrium analyses indicated that the adsorption process was best described by the pseudo-second-order kinetic model and Langmuir isotherm. Overall, the incorporation of xanthan gum improved the adsorption and biological properties of HAp, demonstrating the potential of HAp-XAn as a multifunctional material for dye removal and biomedical applications.

## 1. Introduction

Hydroxyapatite (HAp, Ca_10_(PO_4_)_6_(OH)_2_), a naturally occurring calcium phosphate, is the primary inorganic component of bones and teeth. Its high biocompatibility, bioactivity, and adsorption capability make it a versatile material for both biomedical and environmental applications. The HAp structure provides numerous active sites for ion exchange and surface complexation, enabling efficient removal of heavy metals, fluoride, and organic dyes from aqueous solutions. Previous studies have reported that HAp-based adsorbents can achieve removal efficiencies exceeding 90% for methylene blue (MB), a common model dye pollutant [1].

The antibacterial activity of HAp can be achieved through surface modification or hybridization with other functional materials. Metal-doped HAp systems (e.g., Ag- or Zn-modified HAp) have exhibited strong bactericidal effects against *Escherichia coli* and *Staphylococcus aureus*, while polymer-modified HAp coatings are known to inhibit bacterial adhesion and colonization [2,3,4].

In practical wastewater treatment, chemical pollutants and pathogenic microorganisms can coexist in the same contaminated water, particularly in effluents originating from textile, pharmaceutical, and other industrial activities. Conventional treatment strategies often address these contaminants through separate processes, with adsorption or other physico-chemical methods employed for dye removal and disinfection applied as a subsequent step to control microbial contamination. Although sequential treatment can be effective, it increases process complexity, treatment time, energy consumption, and operational requirements. Therefore, the development of a single multifunctional material capable of simultaneously removing dissolved organic dyes and suppressing bacterial growth could simplify the treatment process and improve its overall efficiency. Such an integrated approach is particularly attractive because it combines pollutant removal and microbial control within one treatment step, potentially reducing the need for multiple treatment stages and materials.

Xanthan gum, an extracellular polysaccharide produced by Xanthomonas species, is widely applied in food, pharmaceutical, and biomedical industries due to its non-toxicity, biodegradability, and rheological properties [5]. Chemically, xanthan contains abundant hydroxyl and carboxyl groups capable of interacting with inorganic surfaces [6,7]. When combined with HAp, xanthan can introduce additional functional groups, improve dispersion and porosity, and stabilize the composite structure. Moreover, the polysaccharide component can enhance biocompatibility and provide synergistic effects in adsorption and antimicrobial performance [8].

Although various HAp-polymer composites have been investigated, systematic studies on xanthan-modified HAp combining dye adsorption and antibacterial activity within a single material remain limited. In particular, the simultaneous evaluation of its environmental performance toward dye removal and its antibacterial and cytocompatibility properties has received limited attention. To address this research gap, we developed a multifunctional hydroxyapatite–xanthan (HAp-Xan) composite using a co-precipitation method to combine dye adsorption, antibacterial activity, and cytocompatibility within a single material. The new material was thoroughly characterized by X-ray diffraction (XRD), Fourier-transform infrared spectroscopy (FTIR), X-ray photoelectron spectroscopy (XPS), scanning electron microscopy (SEM) and atomic force microscopy (AFM) analysis. Its adsorption capacity was assessed using methylene blue as a model dye, with kinetic and isotherm studies performed to elucidate the mechanism. In parallel, antibacterial activity was evaluated against representative Gram-positive and Gram-negative strains.

The integration of adsorption and antimicrobial properties within a single biopolymer-modified HAp system demonstrates the multifunctionality of HAp-XAn. This dual capability positions the composite as a promising candidate for wastewater purification, while its biocompatibility and antibacterial effects also support potential biomedical applications.

## 2. Materials and Methods

### 2.1. Materials and Methods

All chemicals used were of analytical grade. Calcium nitrate tetrahydrate (Ca(NO_3_)_2_·4H_2_O, ≥99%) and diammonium hydrogen phosphate ((NH_4_)_2_HPO_4_, ≥99%) were purchased from Sigma-Aldrich (St Louis, MO, USA) and employed as calcium and phosphate precursors for hydroxyapatite synthesis. Xanthan gum (food/pharmaceutical grade, Sigma-Aldrich, St Louis, MO, USA) was used as the modifying biopolymer. Sodium hydroxide (NaOH, 0.1 M), hydrochloric acid (HCl, 0.1 M), glacial acetic acid, and aqueous ammonia ((NH_3_)aq, 28% *w*/*w*) were utilized for pH adjustments during synthesis. All solutions were prepared using deionized water with a resistivity of 18.2 MΩ·cm.

### 2.2. Synthesis of HAp and HAp-XAn

HAp was synthesized using a controlled aqueous co-precipitation route. A 250 mL solution of calcium nitrate tetrahydrate (Ca(NO_3_)_2_·4H_2_O) was slowly introduced into a diammonium hydrogen phosphate ((NH_4_)_2_HPO_4_) solution while maintaining the stoichiometric Ca/P molar ratio at 1.67. The reaction was conducted under a nitrogen (azote) inert atmosphere to avoid carbonate incorporation, and the pH was adjusted above 10 by the gradual addition of aqueous ammonia ((NH_3_)aq). The mixture was continuously stirred for 4 h and aged for 72 h at ambient temperature. The resulting precipitate was filtered, rinsed with deionized water to neutral pH, dried at 80 °C overnight, and finely ground to obtain pure hydroxyapatite powder.

The HAp-XAn composite was prepared following the same synthesis pathway, except that xanthan gum powder (10 wt% relative to the calcium precursor) was dispersed in the calcium nitrate solution before the phosphate addition. The incorporation of xanthan facilitated the in situ growth of HAp crystals within the biopolymer matrix under identical nitrogen and pH conditions. After precipitation, the material was subjected to filtration, thorough washing, drying at 80 °C, and gentle grinding to yield a homogeneous composite powder.

### 2.3. Characterization Techniques

#### 2.3.1. X-Ray Diffraction (XRD)

The crystalline structure of HAp and HAp-XAn composites was analyzed using a Bruker D8 Advance diffractometer (Bruker, Karlsruhe, Germany) equipped with a LynxEye™ detector and using CuKα radiation (λ = 1.5418 Å). Patterns were collected over a 2θ range of 10–70°, with a step size of 0.02° and an acquisition time of 5 s per step. Lattice parameters were calculated using Equation (1):

(1) $$\frac{1}{d^{2}}=\frac{4}{3}\left[\frac{h^{2}+k^{2}+hk}{a^{2}}\right]+\frac{l^{2}}{c^{2}}$$

The unit cell volume was determined from Equation (2)

(2) $$V=\frac{3\sqrt{3}}{2}a^{2}c$$

Crystallite size was estimated using the Scherrer equation:

(3) $$D_{hkl}=\frac{K\lambda \;}{\beta_{hkl\;}cos\theta_{hkl}}$$

where *D* is the crystallite size, *K* is the shape factor (0.94), *β* is the FWHM of the diffraction peak, *θ* is the Bragg angle, and *λ* is the X-ray wavelength.

#### 2.3.2. Fourier-Transform Infrared Spectroscopy (FTIR)

FTIR spectroscopy was performed to identify functional groups and structural changes induced by xanthan incorporation. Spectra were obtained using a PerkinElmer spectrometer (PerkinElmer, Waltham, MA, USA) with a Diamond/KRS-5 ATR accessory in the range of 400–2000 cm^−1^ at a resolution of 4 cm^−1^. Spectral deconvolution was carried out in the 450–750, 800–1200, and 1200–1480 cm^−1^ regions following established protocols [9].

#### 2.3.3. X-Ray Photoelectron Spectroscopy (XPS)

Surface chemical composition and bonding states were investigated using a SPECS spectrometer (SPECS, Berlin, Germany) with a PHOIBOS 150 analyzer and an XR-50M Mg anode source (E_x_ = 1253.6 eV, 300 W). Charge compensation was applied using a flood gun. Survey spectra were recorded at 50 eV pass energy, and high-resolution spectra at 20 eV. Binding energies were calibrated against the C 1s peak at 284.8 eV. Data were analyzed with CasaXPS software (v2.3.14).

#### 2.3.4. Scanning Electron Microscopy (SEM)

The morphology of the HAp and HAp-XAn powders was observed using a Quanta Inspect F scanning electron microscope (FEI Company, Hillsboro, OR, USA). Samples were examined without conductive coating to preserve surface features.

#### 2.3.5. Atomic Force Microscopy (AFM)

The surface morphology of the HAp and HAp-XAn pellets was investigated by atomic force microscopy (AFM) using an NT-MDT NTEGRA Probe Nano Laboratory instrument (NT-MDT, Moscow, Russia). The measurements were performed in a semi-contact mode at room temperature over a scanning area of 5 × 5 µm^2^. The AFM analysis was used to examine the morphological characteristics of the pellet’s surfaces. Information about the topographical uniformity and the presence of surface irregularities was also obtained. Furthermore, the surface roughness of the pellets was quantified using the root mean square roughness parameter, R_RMS_. The acquired topographical scans and all corresponding data were processed and analyzed with Gwyddion software (v2.59, Czech Metrology Institute, Brno, Czech Republic) [10].

### 2.4. In Vitro Biological Test

#### 2.4.1. MTT Assay

The in vitro cytotoxicity of HAp and the HAp-XAn composites was assessed using MG63 cells with the aid of an MTT assay following a procedure previously described in [11]. For this purpose, the MG63 cells (ATCC CRL1427, American Type Culture Collection, ATCC, Manassas, VA, USA) were cultured in Dulbecco’s Modified Eagle’s Medium (DMEM) and maintained in a humidified incubator at a temperature of 37 °C under an atmosphere of 5% CO_2_. The experiments were performed with cells seeded at a density of 3 × 10^4^ cells/cm^2^ and allowed to adhere before exposure to the tested materials. The cell viability was evaluated after 24, 48, and 72 h of incubation with the HAp and the HAp-XAn composites. At each time point, the culture medium was replaced with medium containing MTT solution at a final concentration of 1 mg/mL, and the cells were incubated for an additional 2 h at 37 °C. After the incubation, the formation of formazan crystals, corresponding to the metabolic activity of viable cells, was quantified by measuring the absorbance at 595 nm using a FlexStation 3 microplate reader. The cell viability was expressed as a percentage relative to the untreated control, which was considered 100%, and the results were presented as mean ± standard deviation (SD).

#### 2.4.2. Antibacterial Activity

The antibacterial activity of HAp and HAp-XAn was evaluated against *Staphylococcus aureus* ATCC 25923 and *Escherichia coli* ATCC 25922. For this purpose, fresh bacterial cultures were prepared in sterile broth and adjusted to approximately 5 × 10^6^ CFU/mL. The bacterial suspensions were incubated with HAp-XAn and HAp, at 37 °C for 24, 48, and 72 h, in order to assess the effect of the incubation time on the antibacterial performance of the tested samples. The bacterial viability was determined for each incubation time, and the results were expressed as log CFU/mL versus time. A positive control (C+), consisting of free bacterial suspension, was used to establish the baseline for the bacterial growth. All the experiments were performed in triplicate, and the results were reported as mean ± SD.

### 2.5. Physicochemical Parameters of Adsorption

Methylene blue (MB), a cationic dye, was selected as the representative pollutant for adsorption studies. A stock solution was prepared in distilled water and calibrated using standard concentrations. Quantification was performed by UV-vis spectrophotometry (SV2100 spectrometer, MAC, Daejeon, South Korea) at 664 nm (λ_max_ of MB), enabling accurate correlation between absorbance and concentration. The effects of pH, contact time, and initial dye concentration on adsorption capacity were systematically investigated. Equilibrium behavior was interpreted using Langmuir, Freundlich, Temkin, and Dubinin–Radushkevich models, while adsorption kinetics were analyzed through pseudo-first- and pseudo-second-order approaches. After adsorption, suspensions were filtered and the residual dye concentration measured. The equilibrium adsorption capacity was calculated from the mass balance equations as follows:

(4) $$q_{e}=\frac{\left(C_{0}-C_{e}\right)V}{m}$$

where q_e_ (mg/g) denotes the adsorption capacity at equilibrium, C_0_ and C_e_ (mg/L) are the initial and equilibrium concentrations of the ion in solution, V (L) the volume of the solution, and m (g) is the mass of the adsorbent.

### 2.6. Adsorption Isotherms of Methylene Blue

Adsorption isotherms provide insight into the relationship between the amount of dye adsorbed and its equilibrium concentration in solution, describing the distribution of molecules on the adsorbent surface. In this study, the equilibrium adsorption of methylene blue (MB) onto HAp and the HAp-XAn composite was analyzed using the Langmuir [12], Freundlich [13], Temkin [14] and Dubinin–Radushkevich (D–R) [15] models:

(5) $$q_{e}=q_{m}k_{L}\frac{C_{e}}{1+k_{L}C_{e}}$$

(6) $$log\left(q_{e}\right)=log(k_{F})\frac{1}{n}log(Ce)$$

(7) $$q_{e}=B\;log(K_{T}C_{e})$$

(8) $${Ln\;q}_{e}={Ln\;q}_{max}-\beta \varepsilon^{2}$$

where q_e_ (mg/g) represents the adsorption capacity at equilibrium, C_e_ (mg/L) is the equilibrium solute concentration, K_L_ (L/mg) is the Langmuir constant, K_F_ is the Freundlich constant adsorption capacity, and n is the heterogeneity coefficient, which describes the degree to which the adsorption process is favorable. B and K_T_ are the Temkin constants, while β is the Dubinin–Radushkevich constant related to the sorption energy and ε is the Polanyi potential.

On the other hand, the Langmuir isotherm can be depicted by a dimensionless constant separation factor (R_L_). The separation factor (R_L_) whose value determines the nature of the isotherm shape is an important feature of the Langmuir isotherm. Also, the R_L_ ratio is sometimes referred to as a dimensionless quantity indicating whether the nature of the adsorption process is favorable (0 < R_L_ < 1), linear (R_L_ = 1) or unfavorable (R_L_ > 1) [16].

The equilibrium parameter is defined as

(9) $$R_{L\;}=\frac{1}{1+k_{L\;}C_{0}}$$

#### Adsorption Kinetics of Methylene Blue

Adsorption kinetics describe the variation in pollutant uptake with time, providing insights into the adsorption mechanism and the rate of transfer at the solid–liquid interface. To investigate the mechanism of MB adsorption onto HAp and HAp-XAn, the experimental data were analyzed using four kinetic models: pseudo-first-order [17], pseudo- second-order [17], intraparticle diffusion [18], and Elovich [18]:

(10) $$log\left(q_{e}-q_{t}\right)={logq}_{e}-\frac{K_{1}}{2.303}t$$

(11) $$\frac{t}{q_{t}}=\frac{t}{K_{2}q_{e}^{2}}+\frac{t}{q_{e}}$$

(12) $$q_{t}=K_{i}t^{0.5}+C$$

(13) $$q_{t}=\frac{1}{\beta }\ln\left(\alpha \beta \right)+\frac{1}{\beta }ln(t)$$

where q_e_ (mg/g) is the adsorption capacity at equilibrium, q_t_ (mg/g) is the adsorption capacity at time t, k_1_ (L·min^−1^) is the rate constant of the pseudo-first-order model, k_2_ (g·mg^−1^·min^−1^) is the rate constant of the pseudo-second-order model, α (mg·g^−1^·min^−1^) and β (mg·g^−1^·min^−1^) are the Elovich constants, and k_i_ is the intraparticle diffusion rate constant.

## 3. Results and Discussions

### 3.1. FTIR Studies

**Figure 1:**
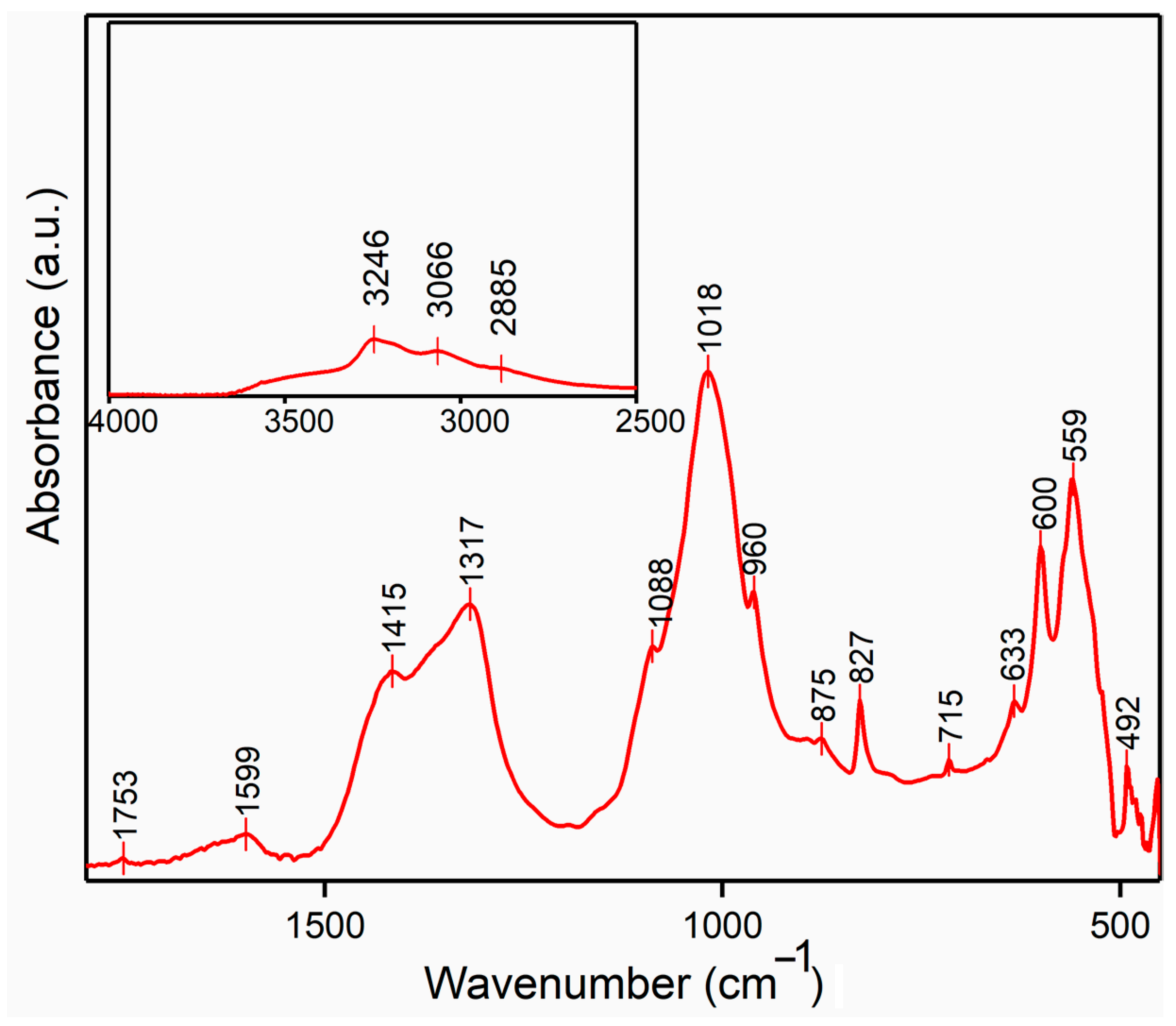
FTIR general spectra of the HAp–XAn samples recorded in the 1800–450 cm^−1^ spectral range. Inset: FTIR general spectra of HAp–Xan in the 4000–2500 cm^−1^ spectral range.

It is well known that hydroxyapatite mainly consists of phosphate and hydroxyl groups, whose characteristic infrared absorption bands define its FTIR spectra. Figure 1 presents the FTIR spectra of HAp-XAn. The HAp FTIR spectra were previously reported in our work [19].

Compared to HAp, the FTIR spectrum of the HAp-XAn composite reveals several spectral changes reflecting the incorporation of the polysaccharide and its interaction with the HAp phase. The characteristic phosphate bands observed in the FTIR spectra of pure HAp were reported in our previous studies [18,19] at approximately 560–600 cm^−1^ (v_4_(PO_4_^3−^)), ~960 cm^−1^ (v_1_(PO_4_^3−^)) and 1030–1090 cm^−1^ (v_3_(PO_4_^3−^)) [20] and are consistent with the one reported in the study conducted by Beigoli, S. and collab [21]. Therefore, these characteristic bands were also observed in the FTIR spectra of the HAp-XAn composite, indicating that the apatite structure was preserved after xanthan incorporation. Moreover, the positions of these phosphate bands (from HAp-XAn composite) were slightly shifted compared with those observed in the FTIR spectra of pure HAp [18,19]. A weak absorption around 875 cm^−1^ is attributed to hydrogen phosphate (HPO_4_^2−^) species from HAp [19]. In the 1200–1000 cm^−1^ spectral region, the maxima attributed to C-O-C and C-O-H vibrations of the xanthan structure are overlapped with the one of phosphate groups from the HAp structure [22]. At the same time, these phosphate bands become slightly broader, suggesting changes in the local chemical environment of the phosphate groups due to organic–inorganic interactions.

The main differences between the two spectra could be noted in the region of 1300–1700 cm^−1^, where additional bands appear only in the HAp-XAn composite. The absorptions centered at approximately 1317 and 1415 cm^−1^ are characteristic of the xanthan polysaccharide and can be attributed to the C–O, C–O–C, symmetrical carboxylate (–COO^−^) stretching vibrations. Moreover, in this spectral region, maxima appear that are characteristic of the bending vibration of carbonate (CO_3_^2−^) groups from HAp [19,23]. Therefore, the carbonate group vibration maxima of HAp could be overlapped with the xanthan bands in this spectral region. The band observed near 1599 cm^−1^ is mainly attributed to the asymmetric stretching vibration of the carboxylate groups, although a contribution from the bending mode of the adsorbed water cannot be excluded. The appearance of these bands confirms the successful incorporation of xanthan into the composite and demonstrates that the organic component remains chemically identifiable after the formation of the composite.

Other differences are observed in the high wavenumber region (2885–3246 cm^−1^), where HAp-XAn exhibits broader absorption features associated with C–H stretching and O–H hydrogen-bonded vibrations [7]. Compared to pure HAp [18,19], the increased width and complexity of this region indicate a broader distribution of hydrogen-bonded environments, which is consistent with the abundance of hydroxyl and C–H stretching vibration in xanthan. Therefore, the FTIR results suggest possible interactions between xanthan and HAp structure. The preservation of the characteristic apatite phosphate bands, together with the appearance of xanthan-characteristic vibrations and the broadening of several absorption bands, suggest the formation of the HAp-XAn composite.

**Figure 2:**
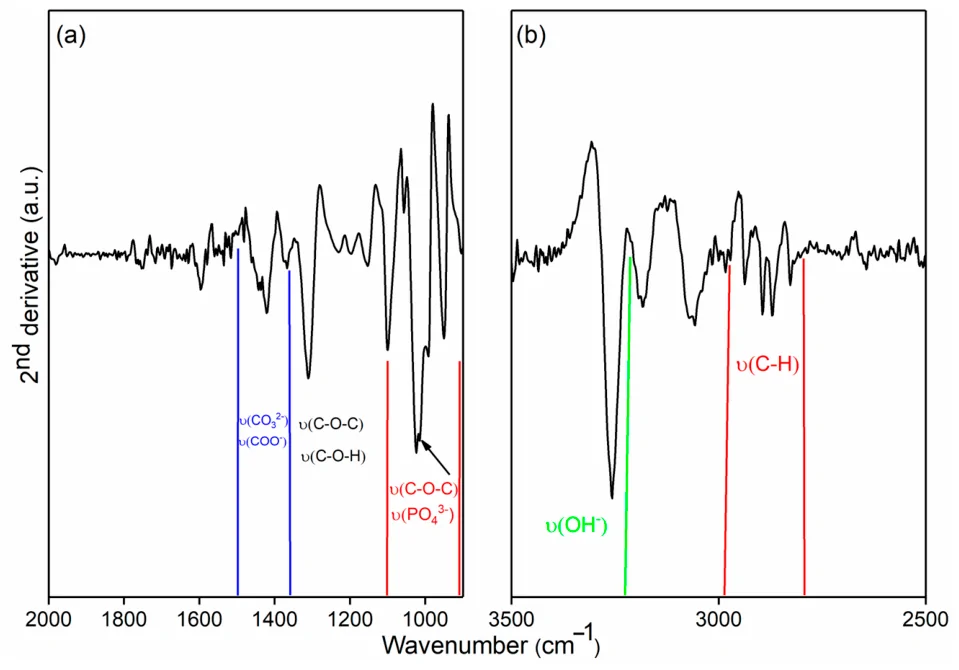
FTIR second derivative spectra of the HAp–XAn sample obtained in the 900–2000 cm^−1^ (**a**) and 2500–3500 cm^−1^ (**b**) spectral domains.

Figure 2a,b reveals the FTIR second-derivative spectra of the HAp–XAn sample obtained in the 900–2000 cm^−1^ and 2500–3500 cm^−1^ spectral domains.

The second-derivative FTIR spectra enhance the resolution of overlapping absorption bands, enabling a more reliable comparison between pure hydroxyapatite (HAp) and the HAp-XAn composite. The results of FTIR second-derivative analysis conducted on the pure HAp sample were reported in our previous work [18]. The second-derivative FTIR spectra of HAp obtained in the 900–1200 cm^−1^ spectral region exhibit maxima corresponding to the phosphate group vibrations, including the v_1_(PO_4_^3−^) symmetric stretching mode at approximately 960 cm^−1^ and the v_3_(PO_4_^3−^) asymmetric stretching components between 1030 and 1090 cm^−1^, which are characteristic of hydroxyapatite (these data were previously reported in our work [18,19]). These phosphate-related features are also observed in the HAp-XAn composite but with slight shifts and band broadening, indicating modifications in the local chemical environment due to the interactions between the mineral phase (HAp) and the polysaccharide matrix (XAn). The bands observed at approximately 1410–1450 cm^−1^ and 1600–1650 cm^−1^ correspond to the symmetric and asymmetric stretching vibrations of carboxylate (–COO^−^) groups, together with contributions from C–O–C and C–O vibrations associated with the polysaccharide backbone [22]. As xanthan is an anionic extracellular polysaccharide rich in hydroxyl, carboxylate, and glycosidic functional groups, the appearance of these characteristic bands confirms its successful incorporation into the composite. Also, in this spectral region, maxima could be observed that are attributed to the carbonate group vibrations [19], which, in the case of HAp-XAn composite, are overlapped with the xanthan-related vibrations.

In the O–H stretching region (2500–3500 cm^−1^), HAp exhibits the broad absorption band typically associated with structural hydroxyl groups and adsorbed water molecules [19]. In contrast, the corresponding second-derivative spectrum of the HAp-XAn composite becomes broader and more complex, indicating the presence of multiple hydrogen-bonding environments. This behavior is consistent with intermolecular hydrogen-bond formation between the hydroxyl and C–H stretching vibration of xanthan and the surface hydroxyl groups of hydroxyapatite, together with possible changes in the coordination state of adsorbed water. These features confirm the successful incorporation of xanthan gum and indicate potential hydrogen and electrostatic interactions between its functional groups and Ca^2+^ sites of the HAp matrix, without disrupting the phosphate framework.

### 3.2. XPS Studies

**Figure 3:**
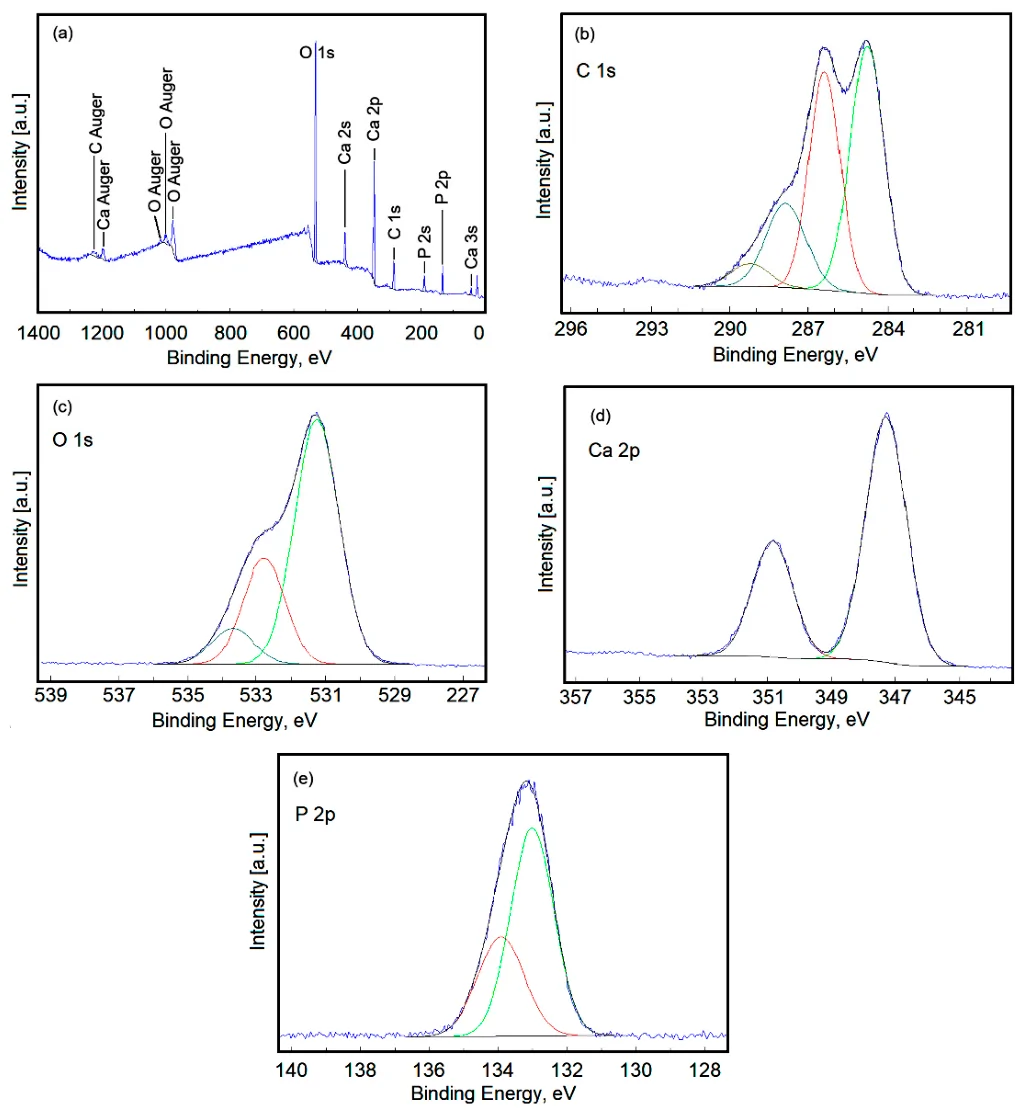
XPS general spectrum of HAp-XAn (**a**) and narrow scan spectra of C (**b**), O (**c**), Ca (**d**), and P (**e**) elements.

The general spectrum of the HAp-XAn composite (Figure 3a) shows peaks associated with the component elements of HAp and xanthan gum (XAn). The high-resolution spectrum of C 1s (Figure 3b) shows four components observed at binding energies (BEs) of 284.80, 286.39, 287.86 and 289.22 eV. The component observed at a BE of 284.80 eV corresponds to C–C single bonds. The component observed at a BE of 286.39 eV is attributed to C–O single bonds characteristic of polysaccharides and also minor surface contaminants. The component identified at a BE of 287.86 eV can be attributed to C=O double bonds or O–C–O. The fourth component located at a BE of 289.22 eV can be attributed to –COOR groups. The XPS high-resolution spectrum of O 1s (Figure 3c) of HAp-XAn composite presents three components. The first component was observed at a BE of 531.26 eV, which was assigned to O in HAp and C=O double bonds. The second component identified at a BE of 532.79 eV can be allocated to C–O single bonds. It is observed that this component increases in intensity due to the presence of XAn. At a BE of 533.69 eV, a third component was observed, which is attributed to adsorbed water and contaminants with C-O single bonds. The high-resolution XPS spectrum of Ca 2p of the HAp-XAn sample (Figure 3d) highlights the characteristic Ca2p doublet with the two specific lines (2p_3/2_ and 2p_1/2_). The Ca2p_3/2_ peak was observed at ~347.29 eV, while the Ca2p_1/2_ peak was observed at ~350.83 eV. The two peaks are spaced at approximately 3.54 eV with an area ratio close to 2:1. This binding energy is attributed to HAp [24]. As can be observed, we have a slight decrease in the binding energy. The xanthan gum has groups such as carboxylate (–COO^−^) derived from glucuronic acid that can interact with Ca^2+^. Since these groups are anionic, they have a high affinity for Ca^2+^. The decrease in the Ca 2p binding energy in HAp-XAn highlights a modification of the chemical environment of Ca^2+^ through interactions with the carboxylate groups of xanthan. This behavior is not due to a structural change in the hydroxyapatite lattice but a result of the HAp surface-biopolymer interaction. The high-resolution XPS spectra of P 2p of the HAp-XAn sample (Figure 3e) show that the presence of two characteristic lines (specific to the P 2p doublet: 2p_3/2_ and 2p_1/2_) could be observed at BEs of 133.02 and 133.90 eV. The peak observed at a BE of 133.02 eV is dominant and is consistent with the phosphate groups (PO_4_^3−^) in hydroxyapatite. The specific lines of the P 2p doublet are spaced at approximately 0.88 eV with an area ratio close to 2:1. The binding energy of the doublet is specific to hydroxyapatite (–PO_4_). As in the case of the peaks associated with O 1s and Ca 2p, the binding energy in the case of P 2p decreases slightly. The decrease in the binding energy of P 2p is also associated with the presence of XAn. As is known, the phosphorus in HAp is located in the PO_4_^3−^ anion, being stable and strongly coordinated in the crystal lattice. As a result, xanthan does not “bond” directly to P. However, there may be secondary interactions between hydrogen bonds with the oxygens in PO_4_^3−^. The hydroxyl groups (–OH) present in xanthan can form hydrogen bonds with the OH groups on the surface of HAp. The modification of the chemical environment leads to an increase in the local electron density and a reduction in the ionic character of the Ca–O–P bonds.

XPS analysis confirmed that the introduction of xanthan gum leads to a change in the surface chemistry of hydroxyapatite. This change that occurs at the surface is evidenced by slight shifts of the binding energies for O 1s, Ca 2p and P 2p towards lower values [19,25]. The XPS results on HAp-XAn reported in this study are in agreement with previous studies, which show that when HAp is modified with biopolymers, the specific peaks Ca 2p, O 1s and P 2p show a constant shift to lower energies. Previous studies [19] showed that for hydroxyapatite, the BEs of XPS peaks for O 1s were located at 531.79, 533.18 and 534.45 eV. For Ca2p, the XPS peaks were assigned at BEs of 347.92 and 351.48 eV, while XPS peaks for O 1s were identified at BEs of 133.36 and 134.23 eV. The present study is also supported by previously reported results [25] regarding the “Development of Zinc-Doped Hydroxyapatite by Sol-Gel Method for Medical Applications” in which it was shown that the specific O1s, Ca 2p and P 2p XPS peaks for hydroxyapatite were observed at BEs of 134.4, 347.4, 350.9, 133.18 and 134.13 eV. This behavior demonstrates the disruption of the Ca–O–P coordination and confirms the formation of a stable HAp-XAn interface, without affecting the basic crystalline structure of apatite. XPS studies highlight an effective interaction between XAn and the HAp surface, with a direct impact on the surface chemical environment.

### 3.3. XRD Studies

**Figure 4:**
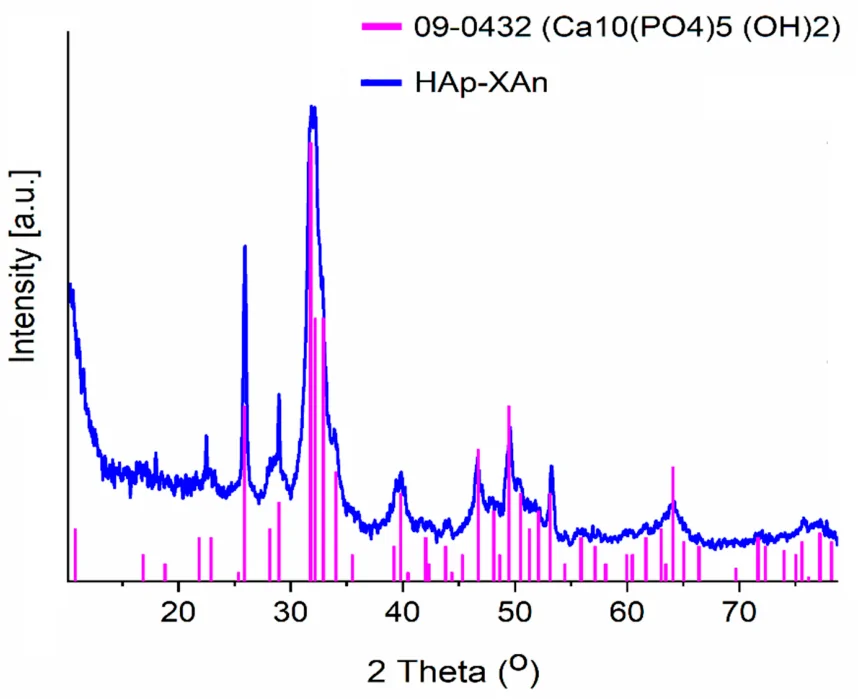
X-ray diffraction pattern of hydroxyapatite–xanthan composite (CuKα radiation). Standard diffraction pattern of hexagonal hydroxyapatite (JCPDS No. 09-0432) is shown in magenta color.

The structural analysis of HAp-XAn composite was performed by powder X-ray diffraction. The XRD patterns of the synthesized HAp-XAn are presented in Figure 4. The XRD pattern of HAp-XAn shows sharper peaks, which indicate better crystallinity. The XRD patterns of HAp–XAn samples revealed diffraction peaks that are in good agreement with the standard hexagonal hydroxyapatite phase, space group P63/m (JCPDS 09-0432) [3]. The peaks observed in the diffraction patterns of HAp-XAn composite are associated with the reflections of the (002), (102), (210), (211), (300), (202), (310), (222), (213) and (004) planes, confirming the formation of crystalline hydroxyapatite. As can be seen, no secondary phases or other impurities were identified. The results are in good agreement with previous results [9].

The incorporation of xanthan gum led to minimal changes in the lattice parameters (“a”, as well as “c”). The “a” and “c” parameters showed a slight expansion compared to pure HAp. The “c/a” ratio remained constant (Table 1). A slight increase in the unit cell volume was also observed (Table 1). The slight increase in the unit cell volume suggests a slight dilation of the hexagonal HAp lattice by introducing defects associated with surface interactions. Local perturbations of the interplanar distances lead to the expansion of the lattice parameters and the unit cell volume compared to the theoretical values without altering the basic crystal structure of HAp.

XRD studies showed that xanthan gum is well incorporated into the final composite without modifying the crystalline structure of hydroxyapatite (the peak positions are identical to those of pure hydroxyapatite with a hexagonal structure). Compared to the hydroxyapatite peaks obtained from synthesis [19], in the case of the HAp-XAn sample, a reduction in peak intensity and a slight broadening of them is observed. Following that, the incorporation of xanthan gum into hydroxyapatite leads to a decrease and broadening of XRD reflections. The incorporation of xanthan gum into hydroxyapatite leads to a reduction in crystallinity and the appearance of microdeformations generated by polymer–surface interactions. This behavior is generated by the –OH and –COOH groups of xanthan that bind to Ca^2+^ and PO_4_^3−^ on the HAp surface. The formation of polymer–surface interactions in HAp-XAn composites is confirmed by FTIR studies by characteristic changes in the –COO^−^, PO_4_^3−^ and OH bands. The coordination of Ca^2+^–COO^−^ is indicated by the shift of the carboxylate vibrations. On the other hand, the broadening and slight shifts of the PO_4_^3−^ bands highlight the disruption of the Ca–O–P lattice. Furthermore, the formation of hydrogen bonds between xanthan and the apatite surface is confirmed by the attenuation and broadening of the specific OH band. As a result of these surface bonds, a better dispersion of the particles is achieved, which induces a decrease in the size of the crystallites and an increase in hydration.

### 3.4. SEM Studies

**Figure 5:**
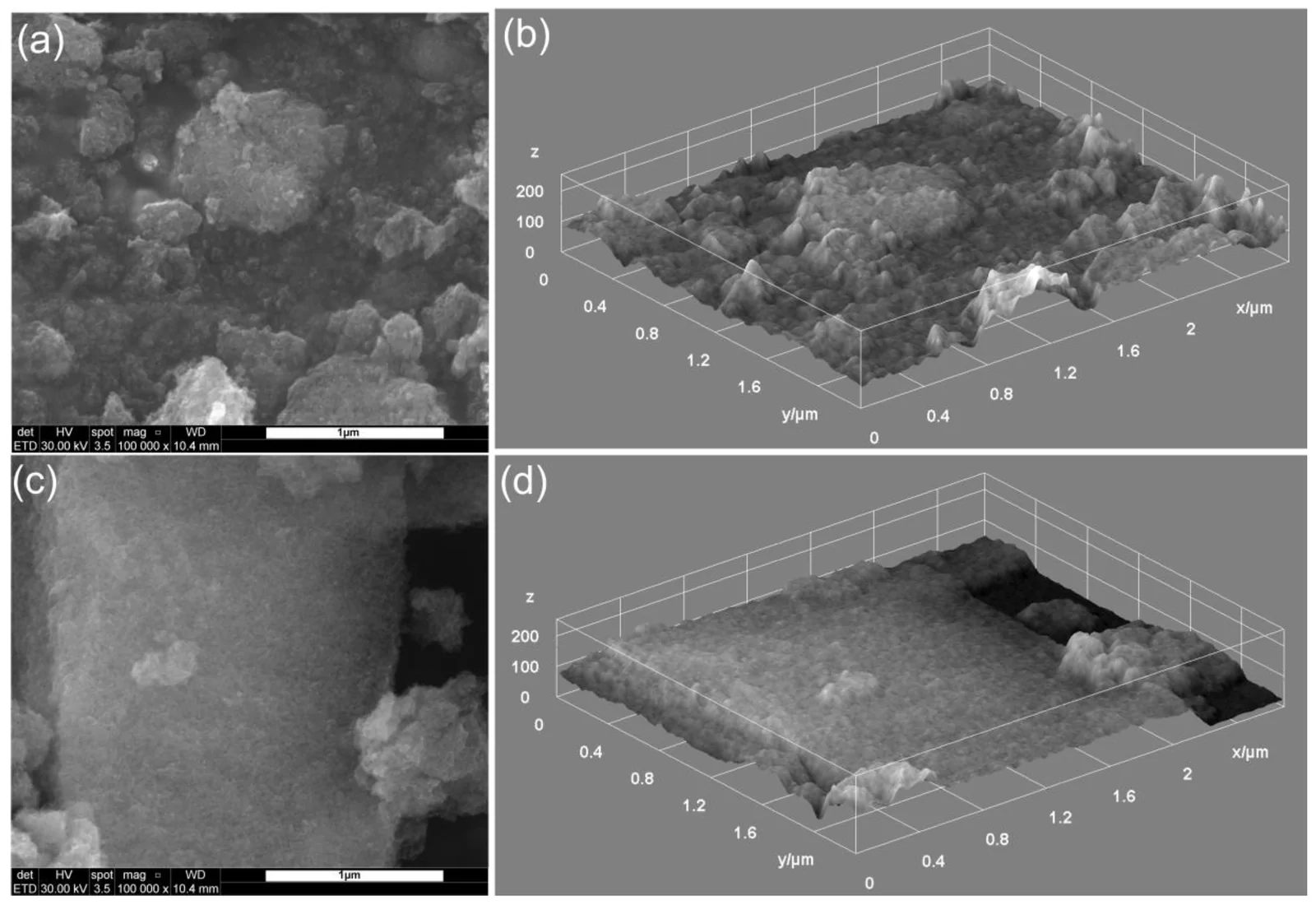
Two- and three-dimensional representations of SEM images of HAp (**a**,**b**) and HAp-XAn (**c**,**d**) samples. 1 µm.

Data about the sample’s morphology were obtained by performing SEM studies. Figure 5 presents SEM images (2D and 3D representations) obtained on the HAp (Figure 5a,b) and HAp-XAn (Figure 5c,d) samples. The SEM images were obtained at a magnification of ×100.000.

Both 3D SEM images (Figure 5b,d) confirmed the characteristics observed in the 2D SEM images of the HAp and HAp-XAn powders. Overall, both 2D and 3D SEM images demonstrate that the incorporation of xanthan changes the morphology and topography of the hydroxyapatite powder and makes the obtained composites suitable candidates for biomedical uses.

### 3.5. AFM Studies

**Figure 6:**
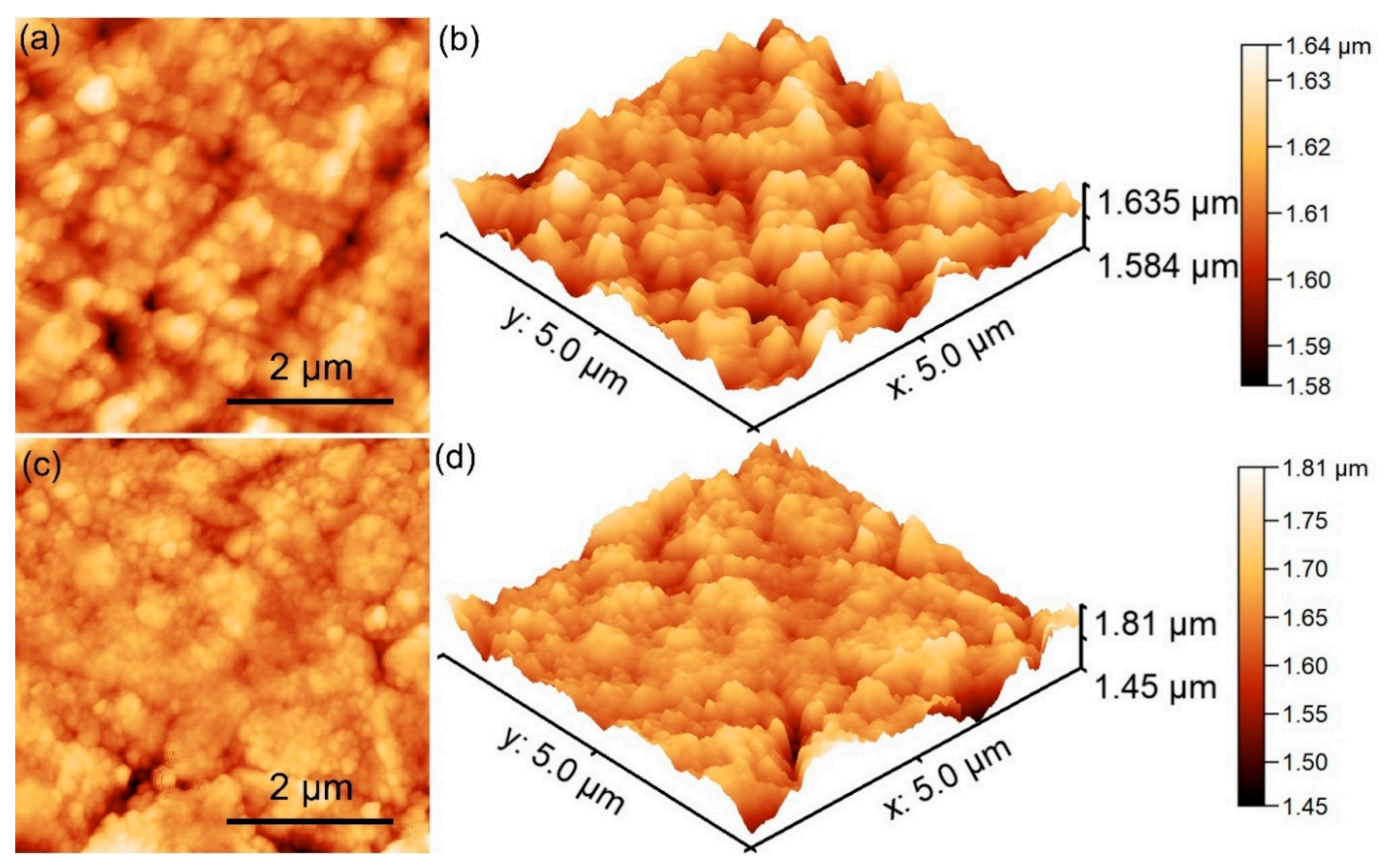
AFM topographic images: (**a**) two-dimensional AFM image of HAp pellet’s surface, (**b**) corresponding three-dimensional topographic reconstruction of HAp pellet’s surface, (**c**) two-dimensional AFM image of the HAp-XAn nanocomposite pellet’s surface, and (**d**) corresponding three-dimensional topographic reconstruction of HAp-XAn pellet’s surface.

Information regarding the surface topography of HAp and HAp-XAn pellets was obtained using the AFM technique. The 2D topographies of the HAp and HAp-XAn surface as well as their 3D representation are depicted in Figure 6a–d. The AFM micrographs of HAp and HAp-XAn revealed a significant difference in the nanoscale surface morphology of the two investigated pellets. The surface topography of the HAp pellet depicted in Figure 6a revealed a relatively smooth and homogeneous topography with particle conglomerates distributed evenly on the surface. Furthermore, the AFM topography of the HAp-XAn pellet surface presented in Figure 6c showed a more irregular surface composed of nanoconglomerates of particles uniformly dispersed on the entire area. More than that, the roughness of the HAp and HAp-XAn pellet’s surface was calculated on the entire 5 × 5 µm^2^ scanned area. The results revealed a measured R_Rms_ value of 6.195 nm for the HAp pellet’s surface and an R_Rms_ value of 34.34 nm in the case of the HAp-XAn pellet’s surface. The increase in roughness indicated that the R_Rms_ value of the HAp-XAn pellet surface was 5.54 times higher than that of the HAp pellet surface. This increase in roughness could be attributed to the fact that the incorporation of xanthan had a significant contribution to the surface structure of hydroxyapatite at the nanoscale. The lower roughness measured for HAp suggests that the surface of the HAp pellet is comparatively compact and uniform, with limited vertical height fluctuations across the entire scanned area. In contrast, the considerably higher R_Rms_ value of HAp-XAn emphasizes a more developed surface morphology characterized by stronger local height variation and a more heterogeneous topographical organization. This increase in surface roughness could be attributed to the presence of xanthan in the composite matrix. Because xanthan is a polysaccharide rich in functional groups that contain oxygen, it can interact with the calcium ions and HAp particles during the composite formation, and could influence the nucleation behavior, particle aggregation, and interparticle packing. These types of interactions due to xanthan presence might lead to the formation of rougher, less uniformly organized, or irregular topographical features rather than a perfectly dense and homogeneous mineral surface [26,27,28].

These findings are well supported by previous studies reported on xanthan with hydroxyapatite composites, where xanthan has been shown to be able to influence particle surface chemistry and nanoscale organization, and in composite scaffolds, to contribute to porous, rough surface morphologies favorable for biological performance [26,27,28]. In their study, Bueno et al. [26] reported that xanthan–hydroxyapatite nanocomposites exhibited a heterogeneous surface with an increased roughness. Also, their findings [26] highlighted that this type of nanoscale organization due to xanthan presence was favorable to the cellular uptake and osteoblast-like cell interaction, thus demonstrating that xanthan does not behave only as a passive matrix component but can actively influence the surface properties of hydroxyapatite. More than that, Deligianni et al. [29] showed in their study on the “Effect of surface roughness of hydroxyapatite on human bone marrow cell adhesion, proliferation, differentiation and detachment strength” that increasing the surface roughness of hydroxyapatite significantly improved the human bone marrow cell adhesion, proliferation, and differentiation. Their results highlighted that hydroxyapatite surfaces with increase roughness provide a more biologically favorable interface for bone-forming cells. Overall, these studies suggest that the incorporation of xanthan can beneficially improve hydroxyapatite surface morphology.

### 3.6. MTT Assay

**Figure 7:**
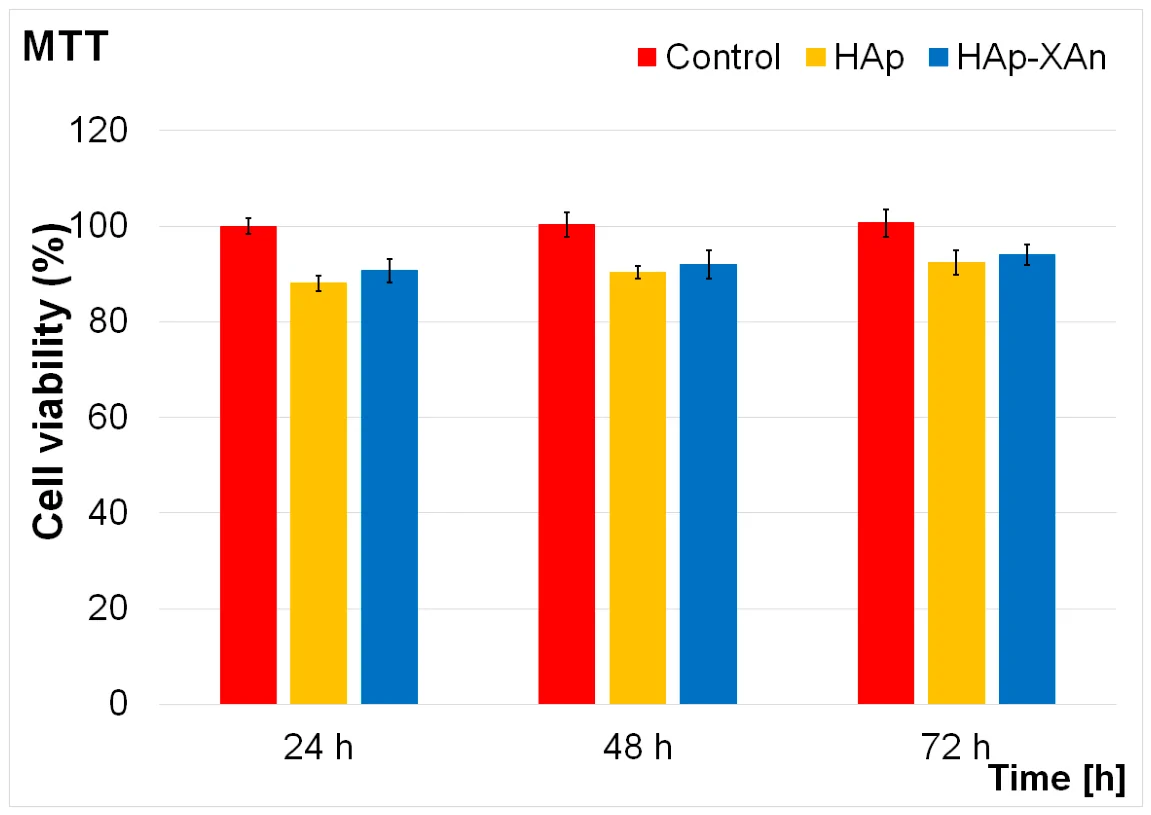
Cell viability of MG63 cells incubated with HAp and the HAp-XAn composites for 24, 48 and 72 h. The results are represented as mean ± standard deviation (SD) and are expressed as percentages of control (100% viability).

The results of the MTT assay are depicted in Figure 7. The findings highlighted that both materials exhibited a favorable biological response throughout the incubation period, confirming their cytocompatible character when exposed to MG63 cells. Furthermore, the data also suggested that the HAp sample had the ability to support a sustained cell survival and metabolic activity over time, indicating that its surface provided a suitable environment for osteoblast-like cell growth.

In the case of HAp-XAn, the results of the MTT assays highlighted that the biological response was more pronounced. The results of the MTT assays showed that cell viability remained high for both HAp and HAp-XAn throughout all the incubation periods. The results highlighted that HAp-XAn presented consistently higher mean viability values than HAp for each incubation period suggesting a more favorable cytocompatibility profile, although these differences were small relative to the standard deviations. Mean viability values also showed a slight increase over the incubation period.

These results could suggest that the MG63 cells were able to adapt, attach, and proliferate efficiently on the composite surface. These findings highlight that the incorporation of xanthan into the hydroxyapatite matrix helped improve the interaction between the cells and materials, most likely by modifying the surface characteristics of the composite in a way that was beneficial for osteoblast-like cells. The higher viability obtained for HAp-XAn in the MTT assay suggests that the composite could be able to provide a more favorable microenvironment for early cellular events than HAp. While HAp is already known for its excellent bioactivity and chemical resemblance to the mineral phase of bone [30,31], the addition of xanthan appears to enhance its overall biological properties [26,27,28,29,30,31,32,33]. The recent review reported by Liu et al. [30] supports the bioactivity and bone-regeneration relevance of HAp, while the study published by Ulian et al. [31] highlights that HAp is chemically related to the mineral phase of bone. The present results emphasize that HAp-XAn combines the intrinsic osteoconductive character of hydroxyapatite with the beneficial matrix-forming and surface-modifying role of xanthan, resulting in improved support for MG63 cell activity. The reported literature data highlighted that the incorporation of xanthan into hydroxyapatite-containing composites could improve the biological performance, cell adhesion, proliferation, and osteoconductive potential, although these effects are formulation-dependent. Bueno et al. [26] reported in their study that xanthan can alter the biological interaction profile of hydroxyapatite-based nanocomposites, suggesting that the incorporation of xanthan could influence how hydroxyapatite behaves in biologically relevant systems rather than acting only as an inert matrix component. These findings are also corroborated by later studies on xanthan-containing hydroxyapatite composites reported by Zia et al. [27] that showed that xanthan-associated composites could improve the biological performance of HA-based scaffolds for bone-related applications. Similarly, studies by Barbosa et al. [32] conducted on chitosan–xanthan gum membranes, incorporating hydroxyapatite for periodontal tissue regeneration, highlighted that the addition of HAp influenced cell adhesion and cell proliferation. Also, the results obtained by Barbosa et al. [32] highlighted that the composite with a higher HA loading showed greater in vitro proliferation of dental pulp mesenchymal stem cells. These findings are well aligned with the results of the MTT assay, further supporting that xanthan-containing HAp composites can exhibit enhanced bioactivity and regenerative potential. Overall, these studies suggest that the addition of xanthan appears to enhance the biological properties of hydroxyapatite-based materials, especially with respect to bioactivity, cytocompatibility, and tissue-regenerative performance.

### 3.7. Antibacterial Assay

**Figure 8:**
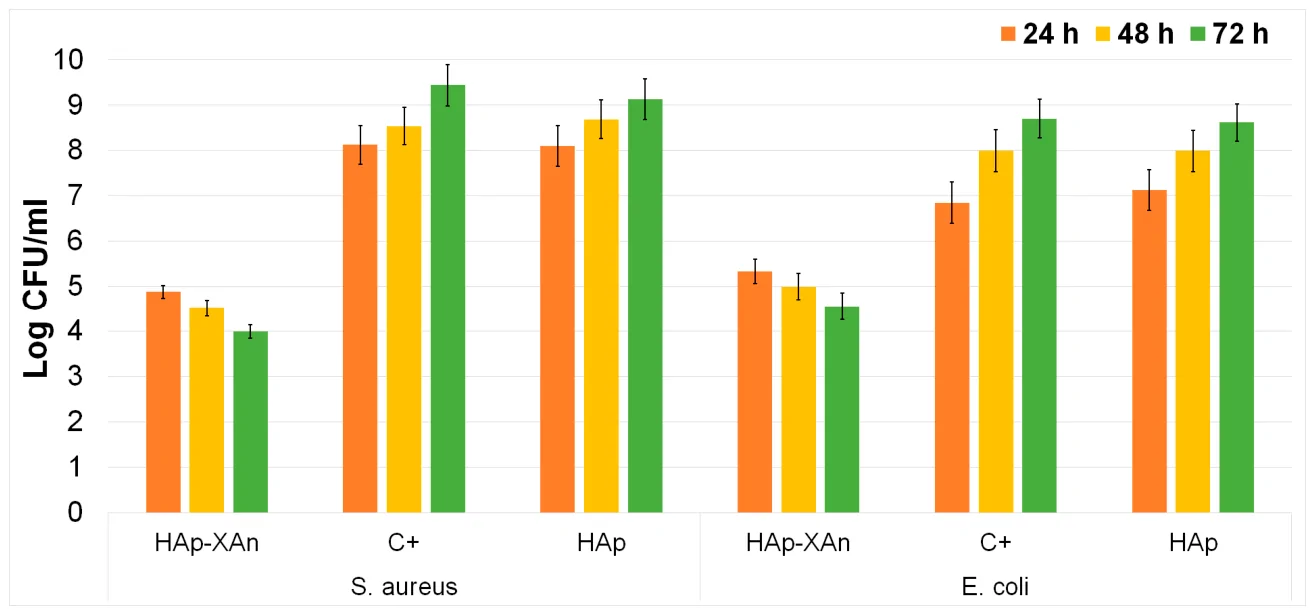
Graphical representation of the log colony forming units (CFUs)/mL of HAp and HAp-XAn incubated with *Staphylococcus aureus* ATCC 25923 and *Escherichia coli* ATCC 25922 for 24, 48, and 72 h. The results are represented as the mean values ± standard deviation (mean ± SD).

The results of the antibacterial assay are depicted in Figure 8. The data suggest that there are noticeable differences in the bacterial survival among the tested samples for both *S. aureus* and *E. coli*. The findings of the in vitro antibacterial assay highlighted that HAp-XAn exhibited the lowest log CFU/mL values for all the tested time periods, suggesting that this sample had a sustained inhibitory effect on the bacterial growth of both tested strains. In the case of *S. aureus* treated with HAp-Xan, a time-dependent gradual decline of the CFU was observed. In contrast, the CFU count for the free bacterial suspension used as a control (C+) increased, while the *S. aureus* treated with HAp exhibited a slight increase in CFU/mL over the same tested period. These results suggest that HAp-XAn was able to inhibit the *S. aureus* growth, while HAp did not prevent the proliferation of the bacterial cells and allowed their increase over time.

Similar results were also obtained in the case of *E. coli.* HAp-XAn had a noticeable inhibitory effect on *E. coli*, while HAp promoted bacterial proliferation. These findings emphasized that HAp-XAn was more effective at limiting bacterial growth than HAp. The time-dependent decrease in CFU/mL in the case of HAp-XAn suggests that its antibacterial action was not only immediate but also maintained or enhanced with the increase in the incubation period. Furthermore, the results of the in vitro antibacterial activity suggest that the incorporation of xanthan may have improved the antibacterial behavior of the material, possibly by enhancing surface interaction, dispersion, or retention of inhibitory properties. On the other hand, the results also highlighted that HAp-XAn exhibited a greater inhibitory effect on *S. aureus* than *E. coli* after 72 h of incubation. The results of the antibacterial assays are in good agreement with previously reported studies regarding the lack or minimum antibacterial properties of HAp and the noticeable inhibitory effects on bacterial strain in the case of xanthan-based composites [33,34,35,36,37,38,39,40,41,42]. The findings highlighted that HAp-XAn showed the higher inhibitory effect on both *S. aureus* and *E. coli* for all tested time periods, while HAp exhibited properties similar to the control. These results could suggest that the antibacterial effect exhibited by HAp-Xan could be attributed to the synergistic effect of the composite components and also to its surface rather than to a strong intrinsic bactericidal activity of hydroxyapatite. This hypothesis agrees well with the reported literature, which generally describes HAp as either weakly antibacterial or without antibacterial properties. In the case of HAp, usually the antimicrobial properties are achieved only after doping or incorporation of antimicrobial agents [33,34,35]. A possible mechanism involved with the HAp-XAn antibacterial properties could be the fact that it can reduce the bacterial survival by limiting the initial adhesion and preventing biofilm formation. Some hydroxyapatite-based materials have been reported to interfere with the bacterial attachment by actively blocking the adhesin-mediated interactions, thus reducing the bacterial surface colonization rather than being direct killing agents [36]. In the case of HAp-XAn, the presence of xanthan could enhance this effect by contributing to the modification of the surface properties of the composite, which are known to strongly influence the bacterial attachment to biomaterials [37,38]. More than that, xanthan could improve the dispersion and stabilization of HAp particles within the composite matrix. Therefore, a more uniform distribution of the mineral could help increase the effective contact area and could lead to a more homogeneous interface for bacterial interaction. Furthermore, xanthan has been shown to act as a structural matrix for hydroxyapatite mineralization, supporting the idea that it can help regulate the composite’s properties and influence its biological performance [39]. On the other hand, xanthan could also be perceived as a physical barrier and could reduce the bacterial adhesion and help slow the formation of bacterial biofilm. Even though xanthan gum is not perceived as being an active antimicrobial agent but rather a functional biomaterial matrix, previous studies have reported that xanthan composites and xanthan derivatives exhibited strong antibacterial activity against *S. aureus* and *E. coli* [40,41,42]. Therefore, in the case of HAp-XAn, the antibacterial activity could be attributed most likely to the fact that xanthan could enhance the antibacterial activity through interfacial and structural synergy rather than acting alone as the main bactericidal agent. Furthermore, the stronger inhibition observed against *S. aureus* than against *E. coli* could also be explained due to a surface-mediated antibacterial mechanism due to the fact that Gram-negative bacteria such as *E. coli* possess an outer membrane that can reduce susceptibility to interfacial disruption, while Gram-positive *S. aureus* is often more vulnerable to changes in surface contact and direct interaction. These findings suggest that the HAp-XAn composite is a promising candidate for the development of future biomedical applications, due to xanthan’s ability to modify HAp properties and support a favorable biological performance.

### 3.8. Effect of pH

The pH of the adsorption medium plays a pivotal role in governing the interaction between the adsorbent surface and the adsorbate species. Variations in pH influence the surface charge of the adsorbent through protonation or deprotonation of active functional groups, thereby modulating the nature and density of adsorption sites. At the same time, the ionization state and molecular speciation of dye molecules are also pH-dependent, directly affecting their electrostatic affinity toward the surface. Consequently, pH control is essential for understanding the underlying adsorption mechanism and optimizing removal efficiency.

In this study, the influence of pH on MB uptake by HAp and HAp–XAn composites was evaluated across a wide pH range (3–11). The solution pH was adjusted using standardized 0.1 M HCl and 0.1 M NaOH solutions. This range encompassed acidic, near-neutral, and alkaline environments, allowing a comprehensive assessment of electrostatic interactions between dye molecules and the adsorbent surfaces.

To isolate the specific effect of pH, all other experimental parameters were held constant: MB initial concentration at 10 mg/L, adsorbent dosage at 12 mg, contact time of 2 h to ensure equilibrium, and temperature fixed at 23 °C. This controlled design provided a clear evaluation of how pH variations affect the adsorption behavior of both pristine hydroxyapatite and xanthan-modified hydroxyapatite.

**Figure 9:**
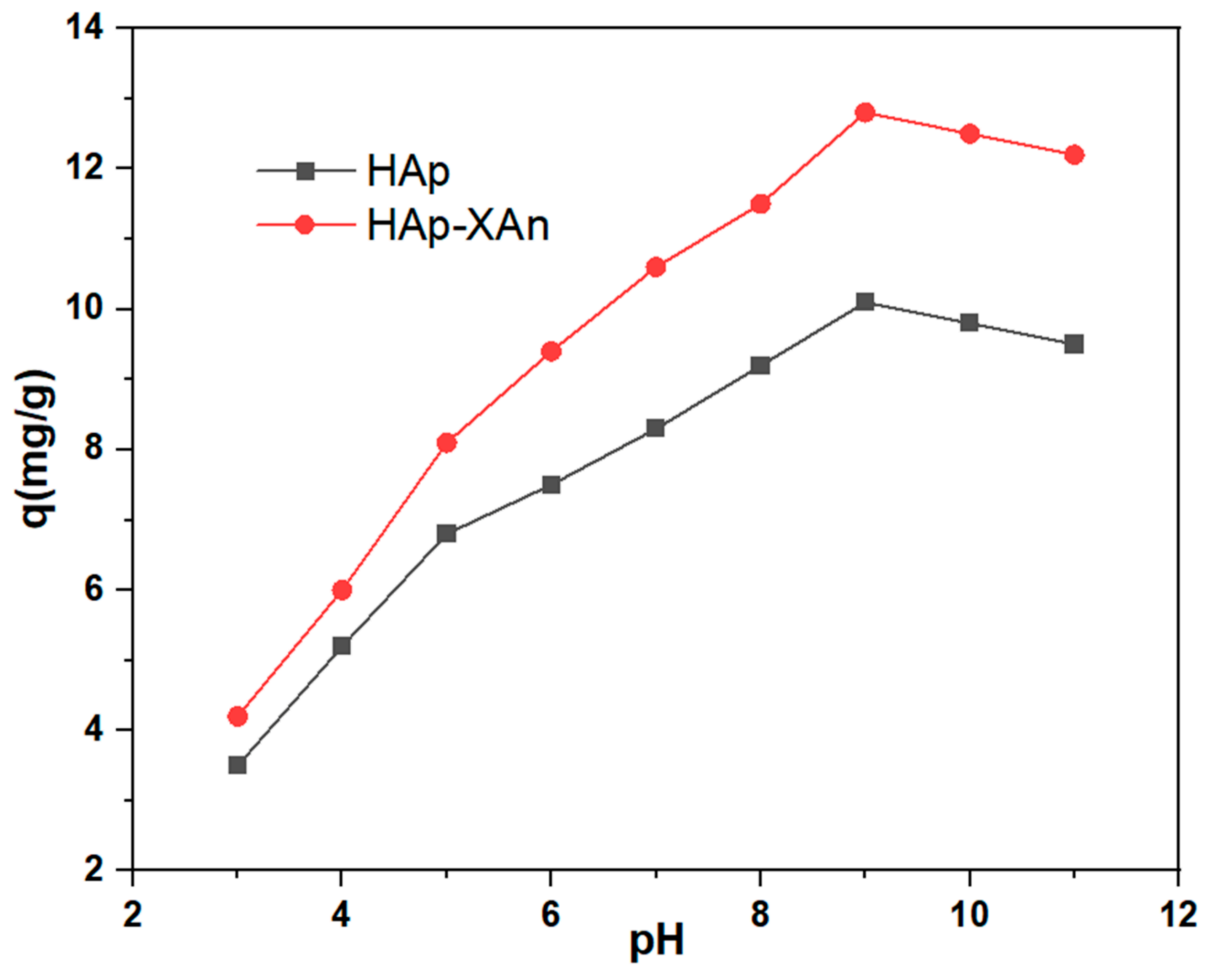
Influence of solution pH on the adsorption of MB by HAp and HAp-XAn.

Solution pH exerted a pronounced influence on the adsorption of MB by both HAp and HAp–XAn composites. The removal efficiency increased progressively with rising pH, with the xanthan-modified material consistently exhibiting superior adsorption compared to pristine hydroxyapatite. The effect of pH on the adsorption of MB by HAp and HAp-XAn in presented in Figure 9. This trend is governed by the surface charge characteristics of the adsorbents. The points of zero charge (pH_pzc_) were determined to be 6.2 for HAp and 7.3 for HAp-XAn, implying that the surface is positively charged below these values and becomes negatively charged in more alkaline media.

At a low pH, the protonation of surface functional groups leads to electrostatic repulsion between the positively charged sites and cationic MB molecules, thereby suppressing adsorption. Conversely, at pH values above the pH_pzc_, deprotonation generates negatively charged surface sites that attract the quaternary ammonium (-N^+^) groups of MB through electrostatic interactions, resulting in enhanced uptake. Consistent with this mechanism, maximum adsorption occurred at pH 9, reaching 10.1 mg/g for HAp and 12.76 mg/g for HAp-XAn. The higher capacity of the composite is attributed to the presence of xanthan gum, which contributes additional -COO^−^ and -OH groups that act as active binding sites and promote stronger electrostatic and hydrogen-bonding interactions with the dye molecules.

### 3.9. Effect of Time

Contact time is a key parameter that determines both the rate and the extent of adsorption, reflecting how rapidly equilibrium between the adsorbent and adsorbate can be achieved. An efficient adsorbent is characterized not only by a high adsorption capacity but also by a short equilibration period. In this study, the adsorption kinetics of MB onto HAp and HAp-XAn composites were investigated by monitoring the adsorption performance over time. The experiments were conducted using 12 mg of adsorbent, an initial dye concentration (C_0_) of 10 mg/L, a solution pH of 5, and a temperature of 24 °C.

**Figure 10:**
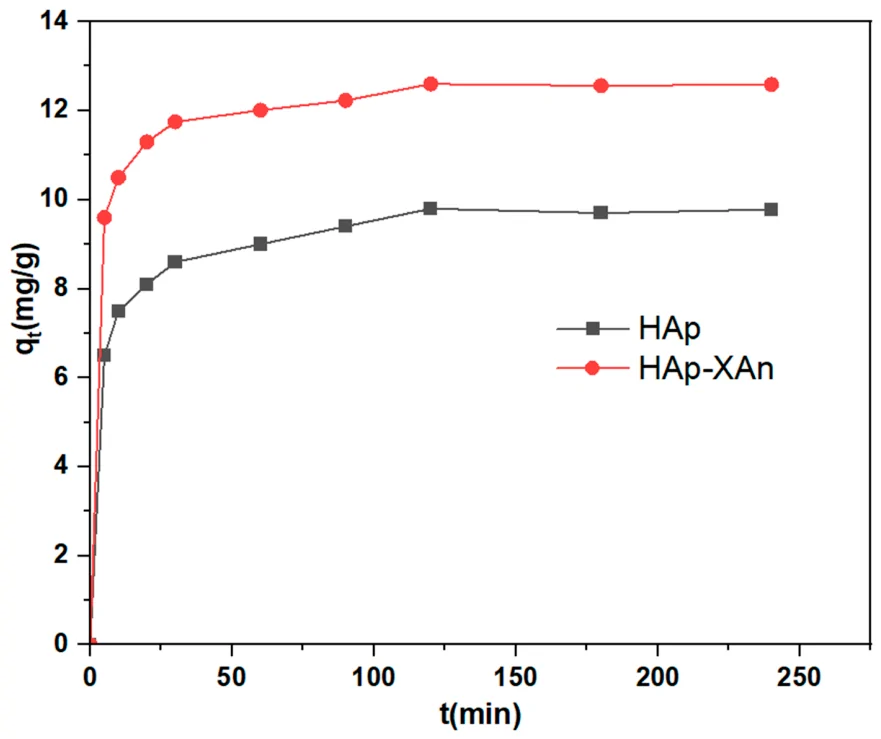
Time-dependent adsorption capacity (q) of MB on HAp and HAp-XAn.

The contact time was varied from 0 to 240 min, during which the amount of adsorbed MB increased progressively with time before reaching equilibrium, as illustrated in Figure 10. The rapid initial uptake is attributed to the abundance of available active sites, while the subsequent plateau indicates saturation of the surface and attainment of adsorption equilibrium.

The adsorption curve of MB on the HAp–XAn composite exhibits two distinct regions. In the initial phase (0–120 min), a rapid increase in adsorption capacity is observed. This fast uptake results from the high concentration of accessible surface sites, including hydroxyl groups from hydroxyapatite and carboxyl or hydroxyl functionalities from xanthan, which readily interact with the dye molecules. During this stage, the steep concentration gradient between the bulk solution and the adsorbent surface provides a strong driving force for mass transfer, promoting efficient diffusion and attachment of MB molecules.

In the subsequent stage (120–240 min), the adsorption rate decreases gradually and the curve levels off, indicating the approach of equilibrium. The slowing rate is attributed to the progressive saturation of active sites and the reduced availability of favorable binding positions. The residual uptake during this period is primarily governed by slower intraparticle diffusion into less accessible pores and weaker interactions with secondary sites. At equilibrium, a dynamic balance is established between the dye molecules adsorbed on the surface and those remaining in solution. This kinetic profile highlights the dominant role of surface functional groups and pore accessibility in controlling the overall adsorption rate and capacity.

### 3.10. Effect of Concentration

The effect of the initial MB concentration on the adsorption behavior of the HAp-XAn composite was investigated within the range of 10–400 mg/L, while keeping all other experimental parameters—contact time, adsorbent dosage, solution pH, and temperature—constant.

**Figure 11:**
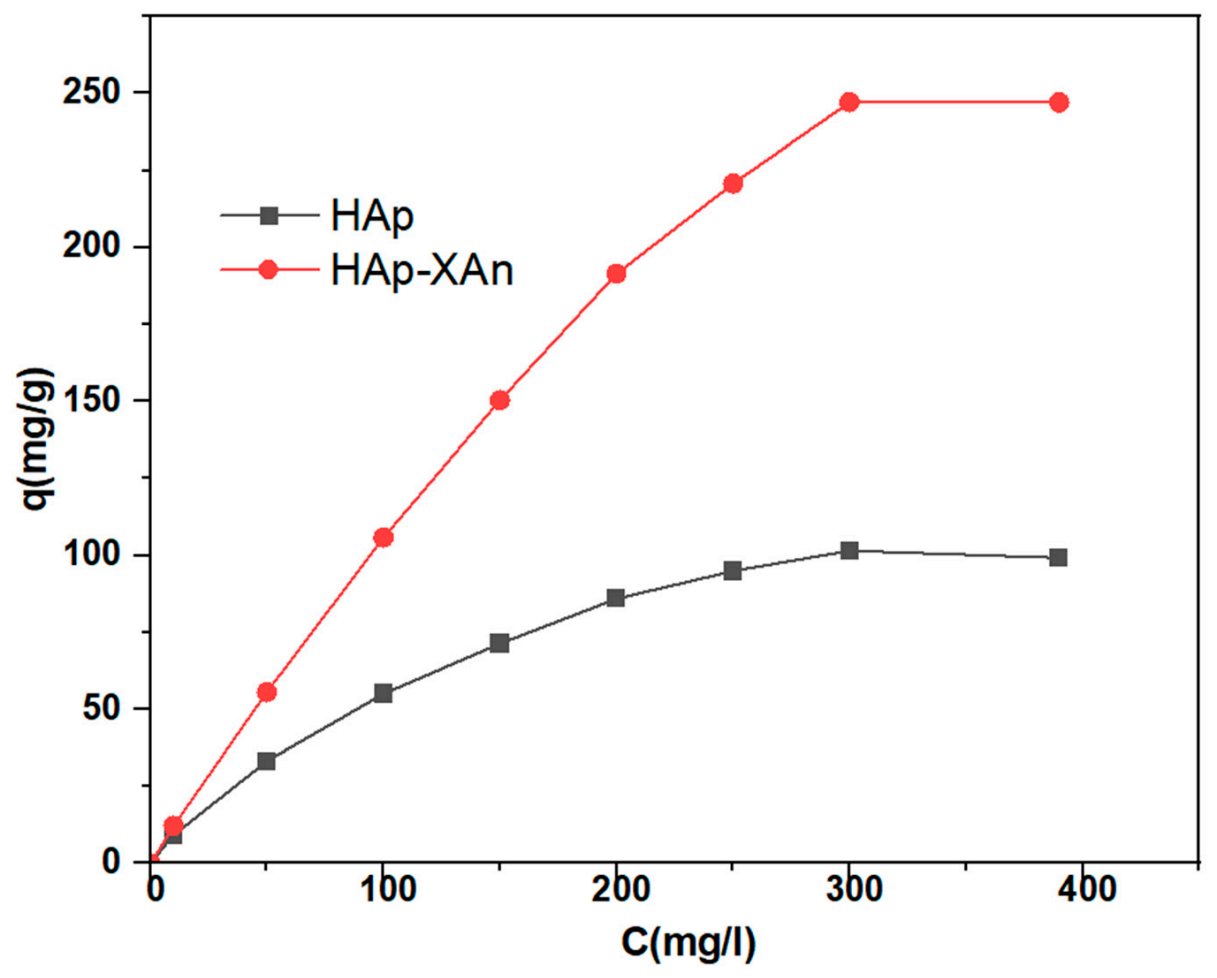
Effect of initial concentration of MB dye adsorption by HAp and HAp-XAn.

In Figure 11, the adsorption results showed that the equilibrium uptake of MB increased progressively with a rising initial concentration. At lower concentrations, numerous unoccupied functional groups on the adsorbent surface enabled rapid dye attachment, yielding efficient but moderate adsorption. As the MB concentration increased, the mass-transfer driving force became stronger, enhancing diffusion toward the surface and intensifying competition among dye molecules for the limited active sites. This led to a marked rise in adsorption capacity until the available surface sites were largely saturated, after which the curve approached a plateau. At higher concentrations, additional increases in MB concentration produced negligible changes in uptake, reflecting surface saturation and possible steric or electrostatic repulsion between adjacent adsorbed molecules.

The consistently higher adsorption capacity of HAp-XAn compared with pure HAp confirms the beneficial contribution of xanthan gum. Its hydroxyl and carboxyl functional groups provide extra binding centers capable of forming hydrogen bonds and electrostatic interactions with cationic dye species, thereby enhancing surface affinity and overall sorption efficiency. The MB adsorption capacity of HAp–XAn was higher than that reported for pristine HAp [43], demonstrating the beneficial effect of xanthan modification. The HAp–XAn composite exhibited a higher MB adsorption capacity than the previously reported CaHAp-(Alg)10 composite (142.850 mg·g^−1^) [44], demonstrating the competitive adsorption performance of the xanthan-modified HAp system.

### 3.11. Isotherm Models

**Figure 12:**
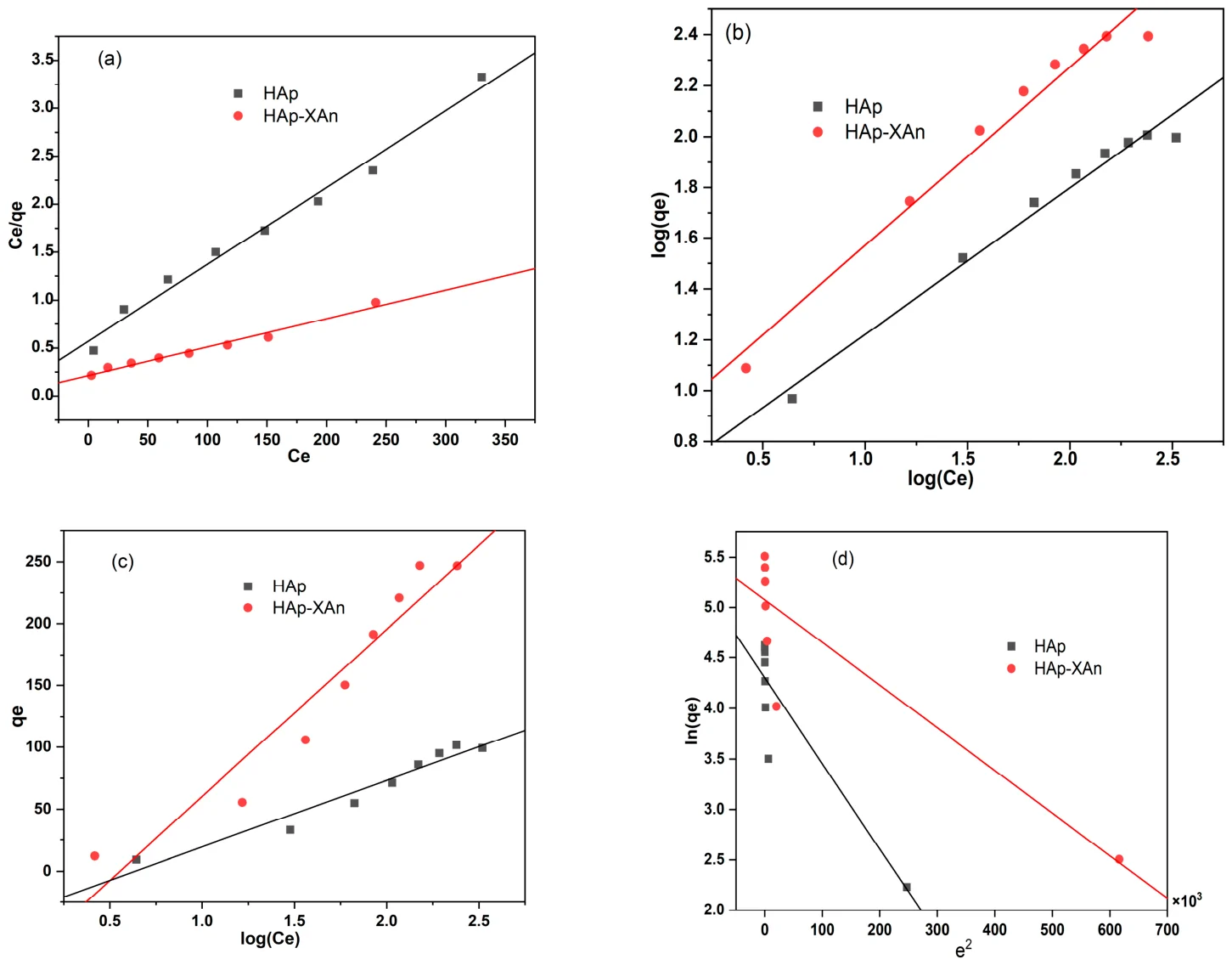
Linearization of the Langmuir (**a**), Freundlich (**b**), Temkin (**c**) and Dubinin–Radushkevich (**d**) equations for adsorbent/adsorbate systems.

Adsorption isotherms provide essential insight into the equilibrium relationship between solute molecules in the liquid phase and those adsorbed onto a solid surface. They describe how adsorbate molecules distribute among the available active sites and reflect the surface characteristics of the adsorbent, such as uniformity, heterogeneity, and the nature of intermolecular interactions. In this work, equilibrium adsorption data for MB were obtained from batch experiments and analyzed using four classical isotherm models—Langmuir, Freundlich, Temkin, and Dubinin–Radushkevich (D-R)—to elucidate the underlying adsorption mechanism and surface behavior of the HAp and HAp-XAn systems, as shown in Figure 12.

The parameters and correlation coefficients (R^2^) derived from the different isotherm models are summarized in Table 2. Among the tested models, the Langmuir equation exhibited the best agreement with the experimental data, as reflected by its highest R^2^ values. The maximum adsorption capacities obtained experimentally—101.55 mg/g for HAp and 247.05 mg/g for HAp-Xan—closely matched the theoretical q_m_ₐ_x_ values predicted by the Langmuir model (125 mg/g and 333.33 mg/g, respectively). Moreover, the R_L_ values ranging between 0 and 1 indicate that the adsorption of dyes onto HAp and HAp-XAn composite is favorable. This strong correlation indicates that methylene blue adsorption occurs predominantly as a monolayer on a uniform surface possessing energetically equivalent sites. Consequently, the Langmuir model provides the most suitable representation of the adsorption behavior of MB onto both pristine hydroxyapatite and the xanthan-modified composite.

### 3.12. Adsorption Kinetics

**Figure 13:**
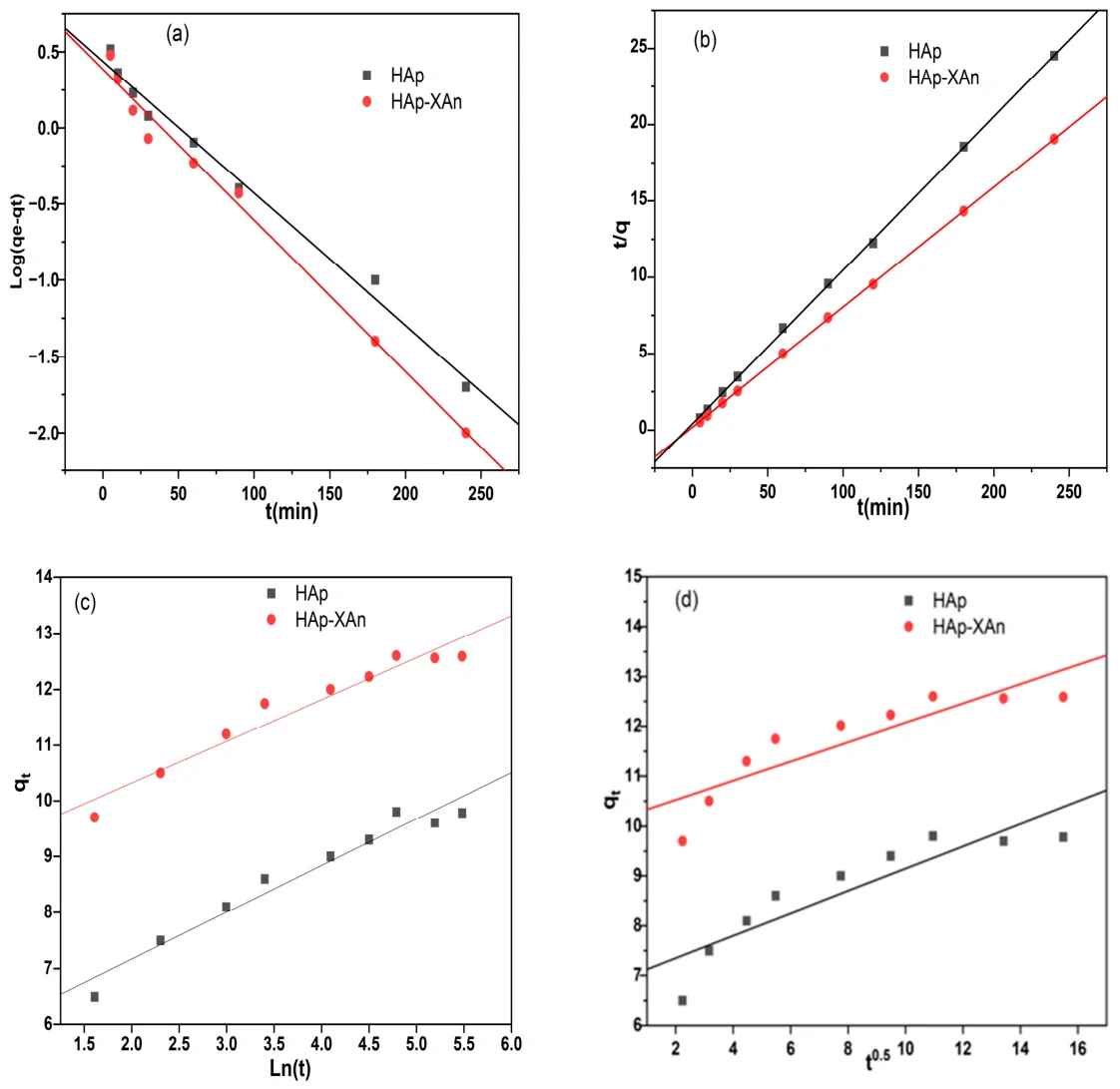
Kinetic modeling of MB adsorption onto HAp and HAp-XAn: pseudo-first-order (**a**), pseudo-second-order (**b**), Elovich (**c**), and intra-particle diffusion (**d**).

The kinetic study of MB adsorption onto HAp and HAp-XAn was carried out using four classical models: pseudo-first-order, pseudo-second-order, intraparticle diffusion, and Elovich. The corresponding kinetic plots are presented in Figure 13.

The applicability of each kinetic model was assessed by comparing the correlation coefficients (R^2^) and the consistency between calculated and experimental equilibrium adsorption capacities (q_e_). As shown in Table 3, the pseudo-first-order, intraparticle diffusion, and Elovich models exhibited weak conformity with the experimental data, reflected by lower R^2^ values (all below 0.869) and significant deviations between theoretical and measured q_e_ values. The results indicate that these models do not accurately capture the adsorption mechanism under the present conditions. In contrast, the pseudo-second-order model provided the best fit for the adsorption of MB onto both HAp and HAp-XAn. This model yielded correlation coefficients approaching unity and calculated q_e_ values that closely matched the experimental measurements. The equilibrium adsorption capacities reached 9.81 mg/g for HAp and 12.61 mg/g for HAp–XAn, confirming the excellent agreement between theoretical and experimental data. Such correspondence demonstrates that the adsorption process follows a pseudo-second-order kinetic pathway, implying that chemisorption governs the overall mechanism. The interaction likely involves valence forces arising from electron sharing or exchange between the functional groups of the adsorbent and the dye molecules.

These kinetic findings not only validate the dominance of the pseudo-second-order mechanism but also highlight the beneficial impact of xanthan incorporation, which enhances dye uptake by introducing additional reactive sites and functional groups. This behavior is consistent with previous studies on polymer-modified hydroxyapatite composites, where pseudo-second-order kinetics have been widely reported as the most accurate model for describing dye adsorption governed by surface chemical interactions.

Correlation of the FTIR, XPS and XRD results confirms that xanthan interacts directly with the hydroxyapatite surface, which leads to a slight change in the surface chemistry and microstructure. XPS highlighted the decrease in binding energies for Ca 2p, O 1s and P 2p, which is characteristic of the interaction of Ca^2+^ with the –COO^−^ groups of xanthan. The Ca/P ratio calculated from XPS was 1.66 for HAp-XAn compared to the stoichiometric value of 1.67 for pure Hap, which reflected changes in surface chemistry rather than bulk composition. This decrease in the Ca/P ratio for HAp-XAn could be due to the interaction between the –COO^−^ and –OH groups of xanthan and superficial Ca^2+^, suggesting that the superficial layer would be apparently deficient in Ca. The results obtained from the XPS studies are supported by those obtained from the FTIR studies, which showed the displacement of the carboxylate vibrations and the broadening of the PO_4_^3−^ bands, highlighting the disruption of the local phosphate environment and the formation of hydrogen bonds between the –OH of the polysaccharide and the HAp surface. On the other hand, the slight increase in the unit cell volume (530.51 Å^3^ versus 528.80 Å^3^ for pure HAp) determined from XRD studies could be due to the appearance of microdeformations and a slight relaxation of the Ca–O–P lattice. The appearance of microdeformations and the slight relaxation of the Ca–O–P lattice could be attributed to the interaction between Ca^2+^ and the carboxylate groups of xanthan and to the local disturbance of the phosphate environment because Ca^2+^ is partially coordinated by xanthan, which leads to changes in the PO_4_^3−^ vibrations (FTIR), the P 2p bond energies (XPS) and the microstructure of the lattice (XRD).

## 4. Conclusions

Hydroxyapatite (HAp) and xanthan-modified hydroxyapatite (HAp–XAn) composites were successfully synthesized via a controlled aqueous co-precipitation method under a nitrogen atmosphere. Structural and spectroscopic analyses confirmed the formation of a single-phase hexagonal apatite, with xanthan incorporation producing no structural alteration but slightly lowering crystallinity and crystallite size. The FTIR spectra showed the presence of phosphate and hydroxyl groups from HAp and of vibrational maxima characteristic of polysaccharides from xanthan, suggesting the interactions between the two components. AFM analysis revealed a noticeable difference in the surface roughness of HAp and HAp-XAn pellets. These findings demonstrate that xanthan could effectively alter HAp surface features and may enhance the interfacial properties of HAp-based biomaterials. The results of the MTT assay demonstrated that both HAp and HAp-XAn possess good cytocompatibility toward MG63 cells for all the tested time intervals. HAp-XAn demonstrated a higher cell viability than HAp, suggesting that xanthan improves the biological properties and cytocompatibility of HAp. Cell viability also increased over time, indicating continued cell adaptation and proliferation. HAp-XAn demonstrated noticeable antibacterial activity against both *S. aureus* and *E. coli* over a 72 h period, in contrast to pure HAp, which exhibited similar activity to the control group. Its antibacterial effect increased over time, indicating time-dependent bacterial inhibition. Overall, the HAp-XAn composite could be considered a promising biomaterial for applications where both bioactivity and infection resistance are required. The incorporation of xanthan substantially improved the methylene blue adsorption performance of HAp, with the adsorption behavior described by the pseudo-second-order kinetic model and Langmuir isotherm.

These results suggest that HAp-XAn could be a promising multifunctional material due to its dye adsorption capacity, antibacterial activity, and cytocompatibility. Further research should evaluate its long-term stability and performance in real wastewater and biological environments.

## Figures and Tables

**Table 1:** XRD analysis results.

Sample	Lattice Parameter (Å)		c/a	Average Crystal Size (nm)	Unit Cell Volume (Å^3^)
	a-Axis	c-Axis			
JCPDS No. 09-432	9.418	6.884	0.731	-	528.80
HAp-XAn	9.432	6.886	0.73	20.37	530.51

**Table 2:** Results obtained using the four models of the adsorption of MB on HAp and HAp-XAn.

Adsorption Models	Parameters	HAp	HAp-XAn
Langmuir	q_max_	125	333.33
	k_L_	0.0375	0.0140
	R_L_	0.7269	0.8765
	R^2^	0.9855	0.9878
Freundlich	K_F_	4.4136	7.4028
	n	1.7340	1.4253
	R^2^	0.979	0.9717
Temkin	K_T_	0.2312	0.2785
	B	53.759	135.4
	R^2^	0.9457	0.9229
D-R	q_max_	73.663	159.748
	E	250	353.553
	R^2^	0.8054	0.7831

**Table 3:** Kinetic parameters of MB adsorption on HAp and HAp-XAn.

Models	Parameters	HAp	HAp-XAn
Pseudo 1st order	q_e_ (exp)	9.81	12.605
	q_e_ (model)	2.739	2.430
	k_1_	0.020	0.02303
	R^2^	0.9881	0.989
Pseudo 2nd order	q_e_ (model)	9.940	12.726
	k_2_	0.0246	0.0326
	R^2^	0.9997	0.9999
Elovich	α	738.2988	130,484.433
	β	1.1999	1.335
	R^2^	0.9567	0.9454
Intraparticle diffusion	K	0.2244	0.1938
	C	6.9025	10.133
	R^2^	0.8162	0.7761

## Data Availability

The data that support the findings of this study are available from the corresponding author upon reasonable request.
